# Quantitative electroencephalographic and neuropsychological investigation of an alternative measure of frontal lobe executive functions: the Figure Trail Making Test

**DOI:** 10.1007/s40708-015-0025-z

**Published:** 2015-11-26

**Authors:** Paul S. Foster, Valeria Drago, Brad J. Ferguson, Patti Kelly Harrison, David W. Harrison

**Affiliations:** 1 Psychology Department, Middle Tennessee State University, 1500 Greenland Drive, Murfreesboro, TN 37132 USA; 2 University of Florida, Gainesville, FL USA; 3 Laboratorio LENITEM, IRCCS San Giovanni di Dio Fatebenefratelli, Brescia, Italy; 4 Behavioral Neuroscience Laboratory, Virginia Polytechnic Institute and State University, Blacksburg, VA USA

**Keywords:** Frontal lobes, Executive functioning, Trail making test, Sequencing, Behavioral speed, Designs, Nonverbal, Neuropsychological assessment, Regulatory control, Effortful control

## Abstract

The most frequently used measures of executive functioning are either sensitive to left frontal lobe functioning or bilateral frontal functioning. Relatively little is known about right frontal lobe contributions to executive functioning given the paucity of measures sensitive to right frontal functioning. The present investigation reports the development and initial validation of a new measure designed to be sensitive to right frontal lobe functioning, the Figure Trail Making Test (FTMT). The FTMT, the classic Trial Making Test, and the Ruff Figural Fluency Test (RFFT) were administered to 42 right-handed men. The results indicated a significant relationship between the FTMT and both the TMT and the RFFT. Performance on the FTMT was also related to high beta EEG over the right frontal lobe. Thus, the FTMT appears to be an equivalent measure of executive functioning that may be sensitive to right frontal lobe functioning. Applications for use in frontotemporal dementia, Alzheimer’s disease, and other patient populations are discussed.

A recent survey indicated that the vast majority of neuropsychologists frequently assess executive functioning as part of their neuropsychological evaluations [1]. Surveys of neuropsychologists have indicated that the Trail Making Test (TMT), Controlled Oral Word Association Test (COWAT), Wisconsin Card Sorting Test (WCST), and the Stroop Color-Word Test (SCWT) are among the most commonly used instruments [1, 2]. Further, the Rabin et al. [1] survey indicated that these same tests are among the most frequently used by neuropsychologists when specifically assessing executive or frontal lobe functioning. The frequent use of the TMT, WCST, and the SCWT, as well as the assumption that they are measures of executive functioning, led Demakis (2003–2004) to conduct a series of meta-analyses to determine the sensitivity of these test to detect frontal lobe dysfunction, particularly lateralized frontal lobe dysfunction. The findings indicated that the SCWT and Part A of the TMT [3], as well as the WCST [4], were all sensitive to frontal lobe dysfunction. However, only the SCWT differentiated between left and right frontal lobe dysfunction, with the worst performance among those with left frontal lobe dysfunction [3].

The finding of the Demakis [4] meta-analysis, that the WCST was not sensitive to lateralized frontal lobe dysfunction, is not surprising given the equivocal findings that have been reported. Whereas performance on the WCST is sensitive to frontal lobe dysfunction [5, 6], demonstration of lateralized frontal dysfunction has been quite problematic. Unilateral left or right dorsolateral frontal dysfunction has been associated with impaired performance on the WCST [6]. Fallgatter and Strik [7] found bilateral frontal lobe activation during performance of the WCST. However, other imaging studies have found right lateralized frontal lobe activation [8] and left lateralized frontal activation [9] in response to performance on the WCST. Further, left frontal lobe alpha power is negatively correlated with performance on the WCST [10]. Finally, patients with left frontal lobe tumors exhibit more impaired performance on the WCST than those with right frontal tumors [11].

Unlike the data for the WCST, more consistent findings have been reported regarding lateralized frontal lobe functioning for the other commonly used measures of executive functioning. For instance, as with the Demakis [3] study, many investigations have found the SCWT to be sensitive to left frontal lobe functioning, although the precise localization within the left frontal lobe has varied. Impaired performance on the SCWT results from left frontal lesions [12] and specifically from lesions localized to the left dorsolateral frontal lobe [13, 14], though bilateral frontal lesions have also yielded impaired performance [13, 14]. Further, studies using neuroimaging to investigate the neural basis of performance on the SCWT have indicated involvement of the left anterior cingulated cortex [15], left lateral prefrontal cortex [16], left inferior precentral sulcus [17], and the left dorsolateral frontal lobe [18].

Wide agreement exists among investigations of the frontal lateralization of verbal or lexical fluency to confrontation. Specifically, patients with left frontal lobe lesions are known to exhibit impaired performance on lexical fluency to confrontation tasks, relative to either patients with right frontal lesions [12, 19, 20] or controls [21]. A recent meta-analysis also indicated that the largest deficits in performance on measures of lexical fluency are associated with left frontal lobe lesions [22]. Troster et al. [23] found that, relative to patients with right pallidotomy, patients with left pallidotomy exhibited more impaired lexical fluency. Several neuroimaging investigations have further supported the role of the left frontal lobe in lexical fluency tasks [15, 24–27]. Performance on lexical fluency tasks also varies as a function of lateral frontal lobe asymmetry, as assessed by electroencephalography [28].

The Trail Making Test is certainly among the most widely used tests [1] and perhaps the most widely researched. Various norms exist for the TMT (see [29]), with Tombaugh [30] providing the most recent comprehensive set of normative data. Different methods of analyzing and interpreting the data have also been proposed and used, including error analysis [13, 14, 31–33], subtraction scores [13, 14, 34], and ratio scores [13, 14, 35].

Several different language versions of the test have been developed and reported, including Arabic [36], Chinese [37, 38], Greek [39], and Hebrew [40]. Numerous alternative versions of the TMT have been developed to address perceived shortcomings of the original TMT. For instance, the Symbol Trail Making Test [41] was developed to reduce the cultural confounds associated with the use of the Arabic numeral system and English alphabet in the original TMT. The Color Trails Test (CTT; [42]) was also developed to control for cultural confounds, although mixed results have been reported regarding whether the CTT is indeed analogous to the TMT [43–45]. A version of the TMT for preschool children, the TRAILS-P, has also been reported [46].

Additionally, the Comprehensive Trail Making Test [47] was developed to control for perceived psychometric shortcomings of the original TMT (for a review see [48] and the Oral Trail Making Test (OTMT; [49]) was developed to reduce confounds associated with motor speed and visual search abilities, with research supporting the OTMT as an equivalent measure [50, 51]. Alternate forms of the TMT have also been developed to permit successive administrations [32, 52] and to assess the relative contributions of the requisite cognitive skills [53].

Delis et al. [54] stated that the continued development of new instrumentation for improving diagnosis and treatment is a critical undertaking in all health-related fields. Further, in their view, the field of neuropsychology has recognized the importance of continually striving to develop new clinical measures. Delis and colleagues developed the extensive Delis-Kaplan Executive Functioning System (D-KEFS; [55]) in the spirit of advancing the instrumentation of neuropsychology. The D-KEFS includes a Trail Making Test consisting of five separate conditions. The Number-Letter Switching condition involves a sequencing procedure similar to that of the classic TMT. The other four conditions are designed to assess the component processes involved in completing the Number-Letter Switching condition so that a precise analysis of the nature of any underlying dysfunction may be accomplished. Specifically, these additional components include Visual Scanning, Number Sequencing, Letter Sequencing, and Motor Speed.

Given that the TMT comprises numbers and letters and is a measure of executive functioning, it may preferentially involve the left frontal lobe. Although the literature is somewhat controversial, neuropsychological and neuroimaging studies seem to provide support for the sensitivity of the TMT to detect left frontal dysfunction [56]. Recent clinically oriented studies investigating frontal lobe involvement of the TMT using transcranial magnetic stimulation (TMS) and near-infrared spectroscopy (NIRS) also support this localization [57]. Performance on Part B of the TMT improved following repetitive TMS applied to the left dorsolateral frontal lobe [57].

With 9–13-year-old boys performing TMT Part B, Weber et al. [58] found a left lateralized increase in the prefrontal cortex in deoxygenated hemoglobin, an indicator of increased oxygen consumption. Moll et al. [59] demonstrated increased activation specific to the prefrontal cortex, especially the left prefrontal region, in healthy controls performing Part B of the TMT. Foster et al. [60] found a significant positive correlation between performance on Part A of the TMT and low beta (13–21 Hz) magnitude (μV) at the left lateral frontal lobe, but not at the right lateral frontal lobe. Finally, Stuss et al. [13, 14] found that patients with left dorsolateral frontal dysfunction evidenced more errors than patients with lesions in other areas of the frontal lobes and those patients with left frontal lesions were the slowest to complete the test.

Taken together, the possibility exists that the aforementioned tests are largely associated with left frontal lobe activity and the TMT, in particular, provides information concerning mental processing speed as well as cognitive flexibility and set-shifting. While some studies have found that deficits in visuomotor set-shifting are specific to the frontal lobe damage [61], others investigators have reported such impairment in patients with posterior brain lesions and widespread cerebral dysfunctions, including cerebellar damage [62] and Alzheimer disease [63]. Thus, it remains unclear whether impairments in visuomotor set-shifting are specific to frontal lobe dysfunction or whether they are non-specific and can result from more posterior or widespread brain dysfunction.

Compared to the collective knowledge we have regarding the cognitive roles of the left frontal lobe, relatively little is known about right frontal lobe contributions to executive functioning. This is likely a result of the dearth of tests that are associated with right frontal activity. The Ruff Figural Fluency Test (RFFT; [64]) is among the few standardized tests of right frontal lobe functioning and was listed as the 14th most commonly used instrument to assess executive functioning in the Rabin et al. [1] survey. The RFFT is known to be sensitive to right frontal lobe functioning [65, 66]; see also [67] pp. 297–298), as is a measure based on the RFFT [19].

The present investigation, with the same intent and spirit as that reported by Delis et al. [54], sought to develop and initially validate a measure of right frontal lobe functioning in an effort to attain a greater understanding of right frontal contributions to executive functioning and to advance the instrumentation of neuropsychology. To meet this objective, a version of the Trail Making Test comprising figures, as opposed to numbers and letters, was developed. The TMT was used as a model for the new test, referred to as the Figure Trail Making Test (FTMT), due to the high frequency of use, the volume of research conducted, and the ease of administration of the TMT. Given that the TMT and the FTMT are both measuring executive functioning, we felt that a moderate correlation would exist between these two measures. Specifically, we hypothesized that performance on the FTMT would be positively correlated with performance on the TMT, in terms of the total time required to complete each part of the tests, an additive and subtractive score, and a ratio score. The total time required to complete each part of the FTMT was also hypothesized to be negatively correlated with the total number of unique designs produced on the RFFT and positively correlated with the number of perseverative errors committed on the RFFT and the perseverative error ratio. We also sought to determine whether the TMT and the FTMT were measuring different constructs by conducting a factor analysis, anticipating that the two tests would load on separate factors.

Additionally, we sought to obtain neurophysiological evidence that the FTMT is sensitive to right frontal lobe functioning. Specifically, we used quantitative electroencephalography (QEEG) to measure electrical activity over the left and right frontal lobes. A previous investigation we conducted found that performance on Part A of the TMT was related to left frontal lobe (F7) low beta magnitude [60]. For the present investigation, we predicted that significant negative correlations would exist between performance on Parts A and B of the TMT and both low and high beta magnitude at the F7 electrode site. We further predicted that significant negative correlations would exist between performance on Parts C and D of the FTMT and both low and high beta magnitude at the F8 electrode site.

## Methods

### Participants

A total of 42 right-handed men, with an age range of 18–29 years (*M* = 20.00, *SD* = 2.10), participated in exchange for extra credit in their undergraduate psychology course. Handedness was assessed using the Coren, Porac, and Duncan Laterality Questionnaire (CPD; [68]), a 13-item questionnaire that assesses lateral preference for the hand, foot, eye, and ear. To be considered for including the participants had to score at least +5 on the CPD (range of scores possible is from –13 to +13, with positive scores indicated increased right-handedness) and identify both biological parents as being right-handed. Further inclusion criteria included having no history of significant head injury or brain dysfunction and no currently experienced psychological problems, as assessed by administering a questionnaire assessing history of head injury, stroke, seizures, paralysis, medical illness, psychological or psychiatric problems, sensory impairments, prescription medication use, and problems with pain or movement.

### Apparatus

#### Ruff Figural Fluency Test

The RFFT [64, 69] is a measure of nonverbal fluency consisting of five individual parts, with each part consisting of a different stimulus pattern. The participants are instructed to draw as many unique designs as possible by connecting at least two of the dots comprising a 5-dot matrix. Nonverbal fluency is then considered the total number of unique designs produced within a 1-min time frame. Other indices of performance included the number of perseverative errors, or the number of instances that any one design is repeated in a single trial, and the perseverative error ratio, or the number of perseverative errors divided by the total number of unique designs.

#### Trail Making Test

The TMT consists of two parts. Part A comprises encircled numbers, 1 through 25, spread in a pseudorandom order across a page. The participant is instructed to draw lines connecting the numbers in order as fast as possible and without picking up the pencil. Part B comprises encircled numbers, 1 through 13, and letters, A through L, spread across a page in a pseudorandom order. The participant is instructed to draw lines alternately connecting the numbers and letters, each in order, as fast as possible and without picking up the pencil. The primary index of performance is typically the time required to complete the test. However, other indices include a subtraction score based on subtracting the time required to complete Part A from the time required to complete Part B as well as a ratio score based on dividing the time required for Part B by the time required for Part A.

#### Figure Trail Making Test

**Fig. 1:**
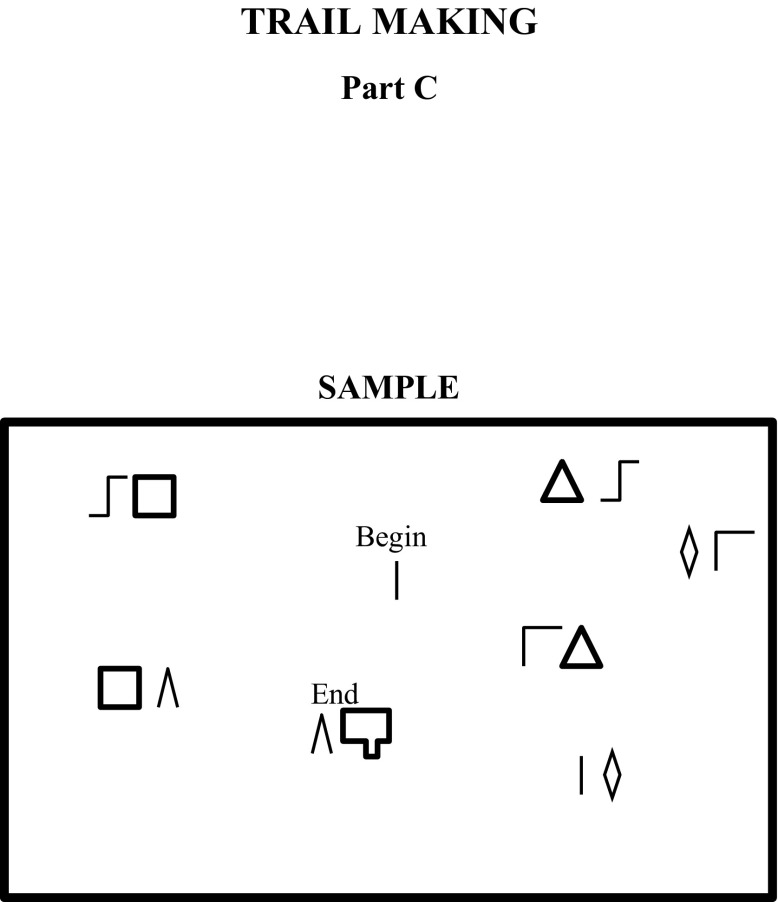
Sample item from FTMT Part C

**Fig. 2:**
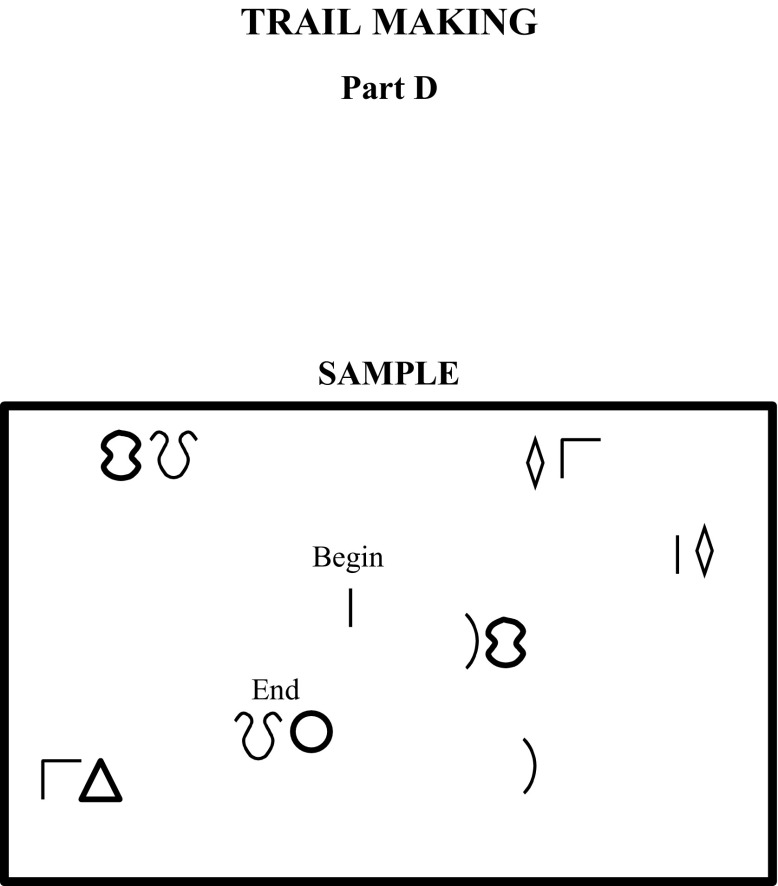
Sample item from FTMT Part D

The FTMT was developed with the intent of preserving the basic principles and format of the TMT. A pseudorandom arrangement of the figures was created by using a vertically inverted mirror image of the original TMT, as has been done with another alternative version of the test [53]. Each part of the FTMT, referred to as parts C and D to help distinguish them from Parts A and B of the TMT, consists of the same number of stimuli as used in each respective part of the TMT. To maintain consistency further between the tests, the first 13 stimuli in Part C of the FTMT were used in the subsequent Part D, just as the numbers 1 through 13 appear in both parts of the TMT. The primary difference between the tests is the use of figural stimuli for the FTMT in the place of numbers and letters as with the TMT. The task involves connecting figure pairs that contain a shared figure. The test begins by locating and drawing a line from an initial single figure to the figure pair that contains the initial figure paired with another new and different figure, which then becomes the target stimulus for the next figure pair. The participant then draws a line from that figure pair to the next pair containing the target figure and another new and different figure. This process continues until the test is completed. The figure that is being sought or the target figure is always located to the left of the figure pair and the new figure is always located to the right of the figure pair. Thus, as with the original TMT, the participant is always aware of the next expected stimulus in the sequence. The set-shifting or switching of Part B of the TMT is accomplished in Part D of the FTMT by having the participant shift between figure pairs comprising angles and figure pairs comprising curves. Each part of the FTMT has an initial sample that the participant completes to familiarize them with the task, just as with the TMT. Figures 1 and 2 present the initial sample for Part C and D of the FTMT, respectively.

#### Quantitative electroencephalography

QEEG was measured using a NeuroSearch-24 (Lexicor Medical Technology, Inc., Boulder, CO, USA). Monopolar QEEG recordings, with linked ear references, were obtained using a lycra electrode cap (Electro-Cap International, Inc., Eaton, OH, USA) containing 19 pure tin electrodes filled with EC2 electrode gel. The electrodes were arranged according to the International 10/20 System. Silver–silver chloride electrodes filled with conductive paste were used for ear references and for measuring electro-oculography. A model 1089 mkII Checktrode Electrode Tester (Lexicor Medical Technology, Inc., Boulder, CO, USA) was used to check the impedance levels of the electrodes.

### Procedure

The participants were initially screened for handedness and history of medical, neurological, and psychological problems. During this initial screening session, the RFFT was administered to groups of 2–8 participants using standard procedures. Following the screening, all participants were invited to participate in the second phase of the investigation, which involved the collection of QEEG data. The participants were given a brief overview of the procedure and were given an opportunity to ask questions. The electrode cap was then affixed to the participant’s scalp using the appropriate anatomical landmarks, followed by the ear references and electro-oculography electrodes. Impedance levels for all of the QEEG electrodes were less than 5 kΩ with most instances being under 3 kΩ. A sampling rate of 256 Hz was used and frequencies below 2 Hz were eliminated by using a high pass filter. The QEEG bandwidth measured included low delta (13.0–21.0 Hz) and high delta (21.0–32.0 Hz). QEEG was sampled for 45 s during an eyes-closed, baseline condition while the participants sat in a sound attenuated chamber. The sampling duration has been standard for this instrumentation, partially due to the file sizes, statistical normality, and precision of measurement with respect to the experimental manipulation(s). Moreover, it has proved effective in prior research from this laboratory. Following collection of QEEG data, the participants were removed from the chamber and the electrodes were removed. The TMT and FTMT were then individually administered to participants. The QEEG data were not recorded concurrent with test administration due to the development of artifacts related to bodily movements and complexity of the behavioral tasks. Standard instructions were used for the TMT (see [29]). The following instructions were used for the FTMT:

On this page (point) are some figures made up of straight lines and angles. Your task is to draw a line connecting the pairs of figures containing the same element. For example, begin here where there is only a single element (point) and draw a line from here to the figure pair that also contains this element (point). Next, draw a line from this figure pair to the figure pair that contains the new element on the right of this figure pair (point). Now, draw a line from this figure pair to the figure pair containing the new element located on the right of the figure pair you are on now (point). Draw another line from the figure pair you are on to the figure pair containing the element on the right (point). The element on the right of each figure pair you are currently on will always be the left element that you are searching for in the next figure pair. No element from any figure pair will be presented more than twice. Keep going in this manner until you reach the end (point). Draw the lines as fast as you can. Ready? Begin!

Similar instructions were given for Part D, with the exception that the participants were instructed to draw a line from an angle figure pair to a curve figure pair and so on. Mistakes made by the participants were corrected, as with the TMT Parts A and B.

## Results

### Data reduction

The raw time required completing each part of the TMT and the FTMT was used in the correlational analyses. Additive, subtraction, and ratio scores were also used in the correlational analyses. Additive scores were obtained by summing the times required to complete the two separate parts of the TMT and the FTMT. Subtraction scores were obtained by subtracting the time required for Part A from that of Part B from the TMT, and Part C from Part D from the FTMT. A ratio score was calculated by dividing the time required for Part B by Part A from the TMT, and Part D by Part C from the FTMT. These alternative scoring procedures were used for exploratory purposes in completion of the statistical analyses based on previous research [13, 14, 35].

### Analyses

**Fig. 3:**
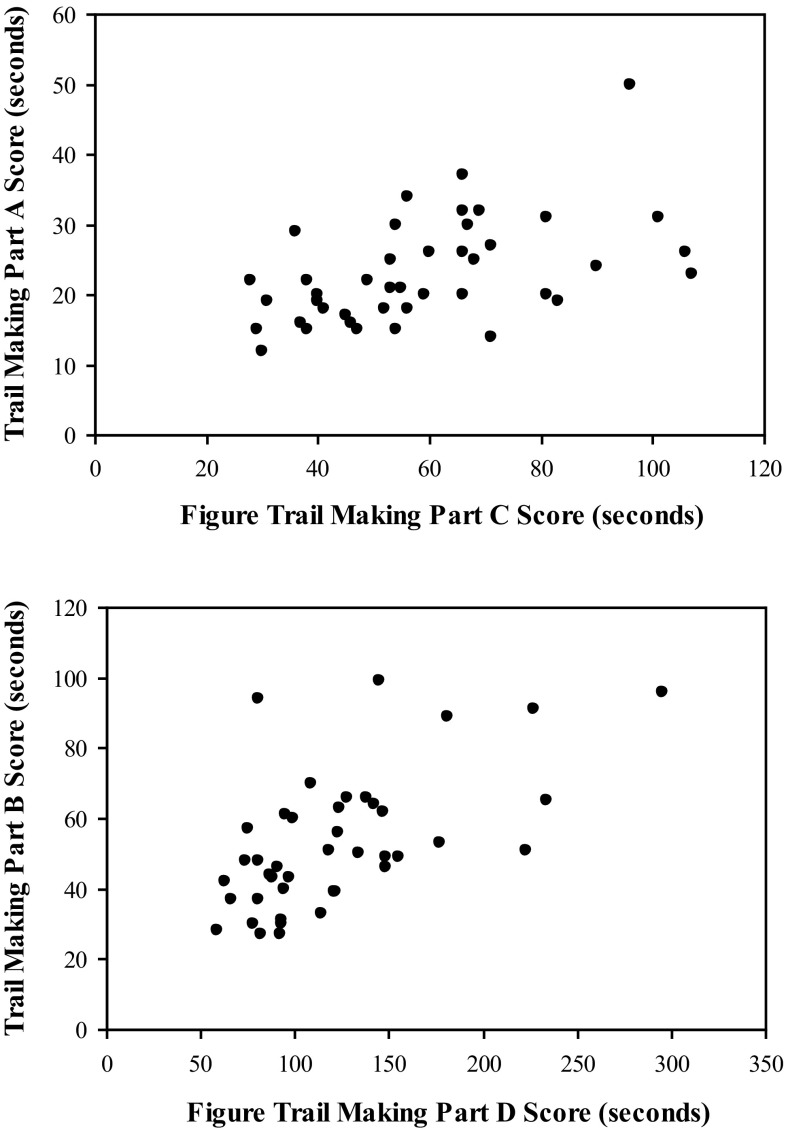
The relationship between TMT Parts A and B and FTMT Parts C and D. Correlational analyses indicated significant positive correlations between the time required to complete TMT Part A and FTMT Part C (*r* = .50, *p* < .001) as well as the time required to complete TMT Part B and FTMT Part D (*r* = .59, *p* < .001)

**Fig. 4:**
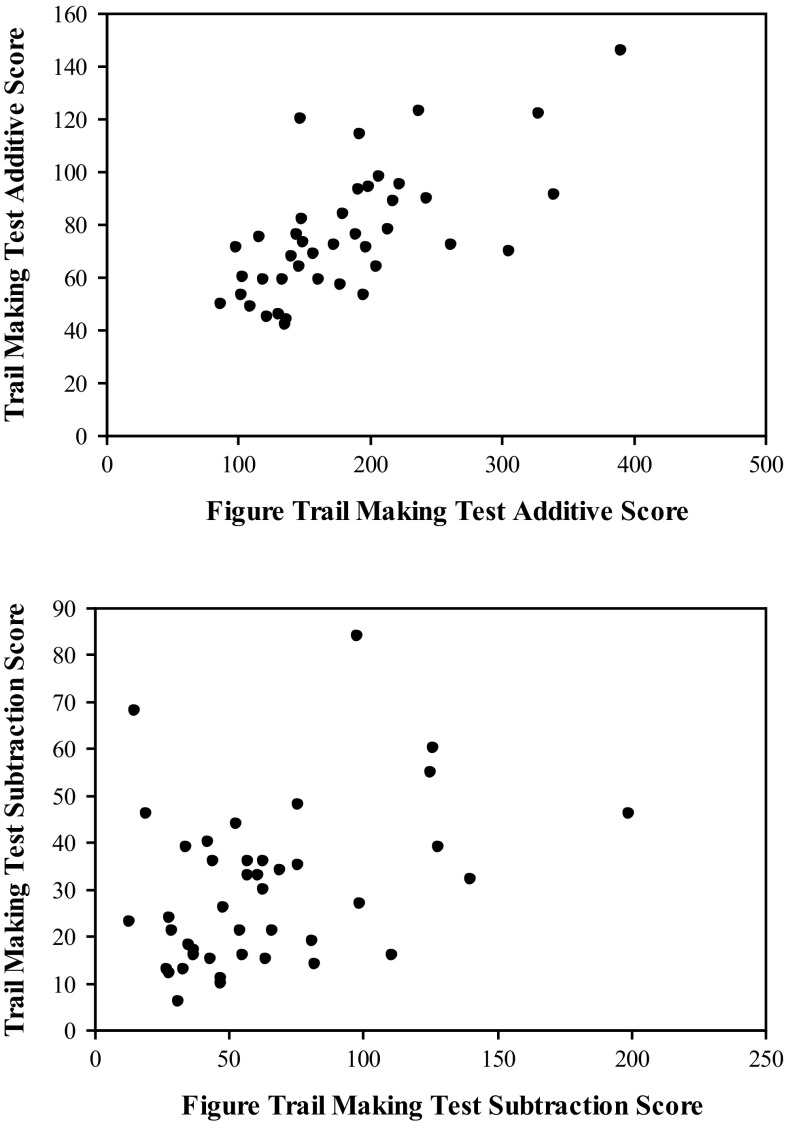
The relationship between the additive and subtraction scores from the TMT and the FTMT. Positive correlations were found between the additive scores from the TMT and the additive score from the FTMT (*r* = .67, *p* < .001), as well as between the subtraction scores from the TMT and the subtraction scores from the FTMT (*r* = .36, *p* = .01)

Means and standard deviations of performance on the RFFT, TMT, and the FTMT may be found in Table 1. Correlational analyses indicated significant positive correlations between the time required to complete TMT Part A and FTMT Part C (*r* = .50, *p* < .001) as well as the time required to complete TMT Part B and FTMT Part D (*r* = .59, *p* < .001; see Fig. 3). Positive correlations were also found between the additive scores from the TMT and the additive score from the FTMT (*r* = .67, *p* < .001), as well as between the subtraction scores from the TMT and the subtraction scores from the FTMT (*r* = .36, *p* = .01; see Fig. 4). The correlation between the ratio scores from the TMT and the FTMT was not significant (*r* = .18, *p* = .12).

**Table 1:** Descriptive statistics for performance on the RFFT, TMT, and the FTMT

	M	SD
Ruff Figural Fluency Test		
Total unique designs	92.76	21.34
Perseverative errors	3.60	2.53
Perseverative error ratio	.039	.026
Trail Making Test		
Part A	23.14	7.46
Part B	52.86	19.25
Additive score	76.00	23.83
Subtraction score	29.71	16.87
Ratio score	2.39	.97
Figure Trail Making Test		
Part C	59.10	21.02
Part D	121.95	51.12
Additive score	181.05	67.97
Subtraction score	62.86	38.62
Ratio score	2.11	.56

**Fig. 5:**
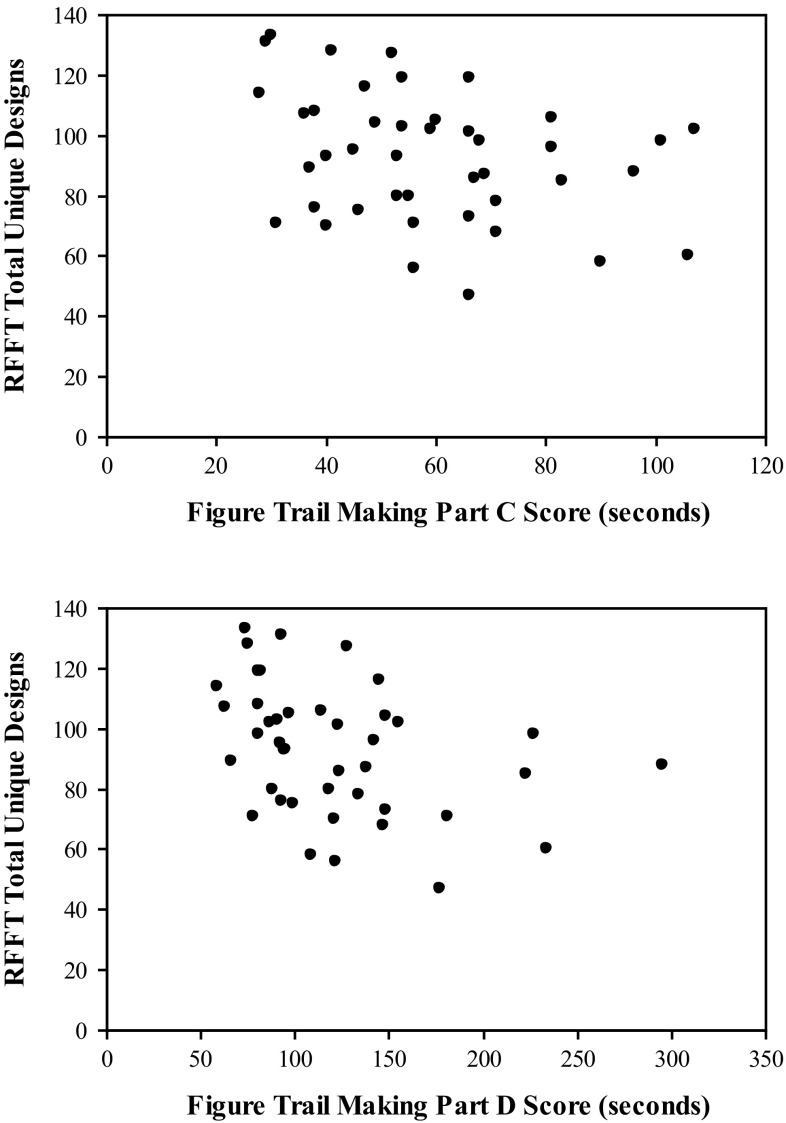
The relationship between FTMT Parts C and D and RFFT total unique designs. Significant negative correlations were found between the total numbers of unique designs produced on the RFFT and both the time required to complete Part C as well as the time required for Part D

**Fig. 6:**
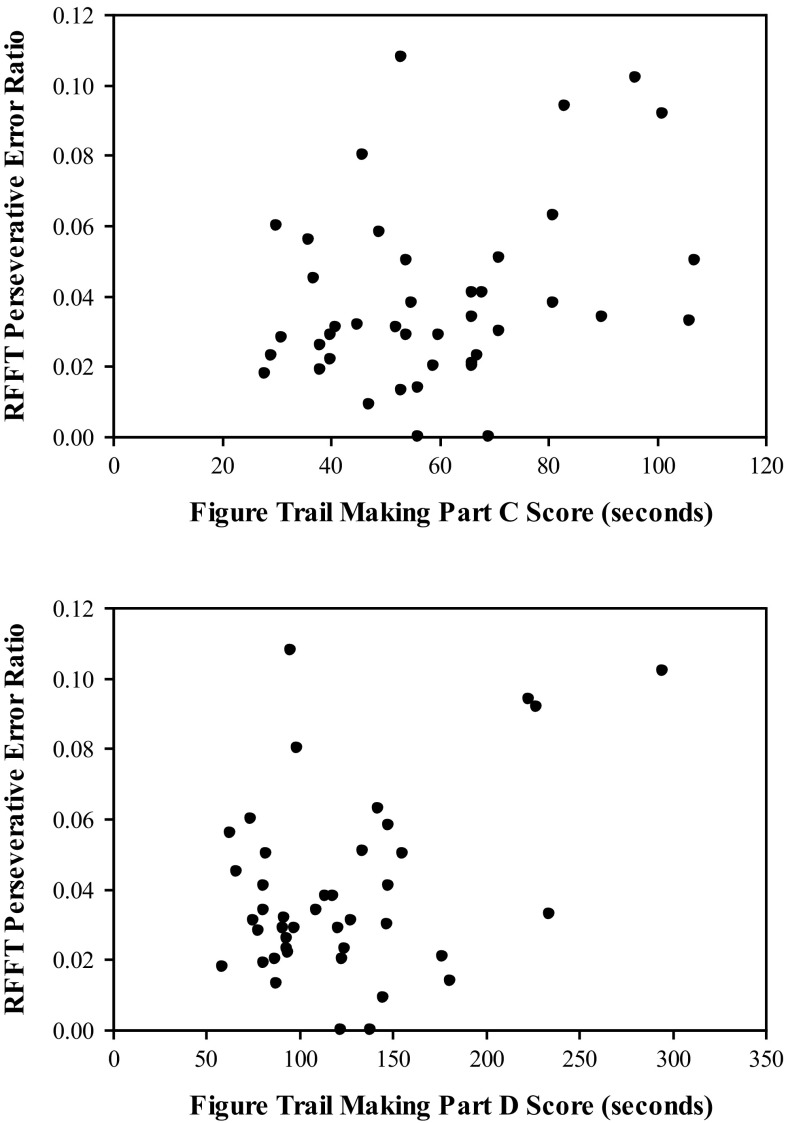
The relationship between FTMT Parts C and D and RFFT perseverative error ratio. Significant positive correlations were found between the perseverative error ratios of the RFFT and Part C as well as Part D

Significant correlations between performance on the FTMT and the RFFT were also found (see Table 2). Specifically, significant negative correlations were found between the total number of unique designs produced on the RFFT and both the time required to complete Part C as well as the time required for Part D (see Fig. 5). Significant positive correlations were found between the perseverative error ratio of the RFFT and Part C as well as Part D (see Fig. 6). No significant correlations were found between the number of perseverative errors on the RFFT and either Part C or Part D.

**Table 2:** Correlation matrix for the Figure Trail Making Test (FTMT) and the Ruff Figural Fluency Test (RFFT)

	RFFT TUD	RFFT PSV	RFFT PER
FTMT Part C	−.32 (.02)	.20 (.10)	.32 (.02)
FTMT Part D	−.37 (.008)	.23 (.07)	.37 (.008)
FTMT Part C + Part D	−.38 (.007)	.24 (.07)	.38 (.007)
FTMT Part D − Part C	−.32 (.02)	.19 (.11)	.32 (.02)
FTMT Part D/Part C	−.10 (.26)	−.02 (.46)	.03 (.44)

Probability reported in parentheses

*RFFT TUD* total unique designs generated on the RFFT, *RFFT PSV* total number of perseverative errors committed on the RFFT, *RFFT PER* the perseverative error ratio for the RFFT

Correlational analyses were also conducted between the original TMT Parts A and B and the RFFT to determine whether any significant relationships existed between these measures. As may be seen in Table 3, a significant negative correlation was found between the total number of unique designs produced on the RFFT and the time required to complete Part A of the TMT. However, no other correlations between any two indices of performance on the TMT and the RFFT were significant.

**Table 3:** Correlation matrix for the Trail Making Test (TMT) and the Ruff Figural Fluency Test (RFFT)

	RFFT TUD	RFFT PSV	RFFT PER
TMT Part A	−.31 (.05)	.04 (.80)	.15 (.34)
TMT Part B	−.05 (.78)	.20 (.21)	.23 (.14)
TMT Part A + Part B	−.13 (.40)	.17 (.28)	.24 (.13)
TMT Part B − Part A	.09 (.59)	.21 (.19)	.20 (.21)
TMT Part B/Part A	.24 (.12)	.15 (.33)	.10 (.52)

Probability reported in parentheses

*RFFT TU* total unique designs generated on the RFFT, *RFFT PSV* total number of perseverative errors committed on the RFFT, *RFFT PER* the perseverative error ratio for the RFFT

The total number of errors and the time to complete each section of the TMT and the FTMT were entered into the factor analysis. Also included in the analysis was the total number of unique designs, number of perseverative errors, and the perseverative error ratio of the RFFT. The results of the factor analysis, with Equamax rotation and Principle Component Analysis for extraction of factors, indicated a five factor solution, collectively accounting for 83.786 % of the variance. The times to complete the two sections of the FTMT, the time to complete Part A of the TMT, and the total number of unique designs produced on the RFFT comprises the first component, accounting for 28.875 % of the variance (3.176 eigenvalue). The second component, accounting for an additional 19.597 % of the variance (2.156 eigenvalue) comprises the number of perseverative errors and the perseverative error ratio of the RFFT. The number of errors and the time to complete Part B of the TMT comprise a third component, accounting for an additional 14.303 % of the variance (1.573 eigenvalue). A fourth component, accounting for another 11.371 % of the variance (1.251 eigenvalue), consists of the number of errors on both sections of the FTMT. Finally, the fifth component consists of the number of errors committed on Part A of the TMT and accounts for an additional 9.640 % of the variance (1.06 eigenvalue). See Table 4 for the component matrix.

**Table 4:** Component matrix from factor analysis

Measure	Component				
	1	2	3	4	5
RFFT TUD	−**.711**	.295	.340	−.112	.220
RFFT PSV	−.045	**.980**	.051	−.112	.075
RFFT PER	.175	**.954**	−.017	−.106	.003
TMT Part A time	**.790**	.017	.126	−.111	.208
TMT Part B time	.476	.154	**.810**	−.026	.101
TMT Part A errors	.073	.027	−.060	.043	**.927**
TMT Part B errors	−.124	−.090	**.942**	.044	−.116
FTMT Part C time	**.752**	.265	.160	.124	.216
FTMT Part D time	**.783**	.314	.269	.306	−.048
FTMT Part C errors	−.015	−.083	.059	**.780**	.330
FTMT Part D errors	.087	−.108	−.035	**.845**	−.187

Significance level established at *p* < .05 shown in bold script

The results from the correlational analyses indicated that no significant correlations existed between low beta magnitude and Parts A and B of the TMT or Parts C and D of the FTMT. Additionally, Parts A and B of the TMT were not significantly correlated with high beta magnitude. However, a significant negative correlation was found between F8 high beta magnitude and performance on Part D of the FTMT. No other significant correlations were found between high beta magnitude and performance on either Part C or Part D of the FTMT (see Table 5).

**Table 5:** Correlations between test performance and high beta magnitude

		Trail Making Test		Figure Trail Making Test	
		Part A	Part B	Part C	Part D
Electrode site	F7	−.08 (.31)	−.17 (.14)	−.15 (.17)	−.17 (.15)
	F8	−.12 (.24)	−.19 (.12)	−.24 (.07)	−.30 (.03)

Probability values reported in parentheses

## Discussion

The need for additional measures of executive functions and especially instruments which may provide implications relevant to cerebral laterality is clear. There remains especially a void for neuropsychological instruments using a TMT format, which may provide information pertaining to the functional integrity of the right frontal region. Consistent with the hypotheses forwarded, significant correlations were found between performance on the TMT and the FTMT, in terms of the raw time required to complete each respective part of the tests as well as the additive and subtraction scores. The fact that the ratio scores were not significantly correlated is not surprising given that research has generally indicated a lack of clinical utility for this score [13, 14, 35]. Given the present findings, the TMT and the FTMT appear to be equivalent measures of executive functioning. Further, the present findings not only suggest that the FTMT may be a measure of executive functioning but also extend the realm of executive functioning to the sequencing and set-shifting of nonverbal stimuli.

However, the finding of significant correlations between the TMT and the FTMT represents somewhat of a caveat in that the TMT has been found to be sensitive to left frontal lobe functioning [13, 14, 57, 59]. This would seem to suggest the possibility that the FTMT is also sensitive to left frontal lobe functioning. The possibility that FTMT is related to left frontal lobe functioning is tempered, though, by the fact that the many of the hypothesized correlations between performance on the RFFT and the FTMT were also significant. Performance on the RFFT is related to right frontal lobe functioning [65, 66]. Thus, the significant correlations between the RFFT and the FTMT suggest that the FTMT may also be sensitive to right frontal lobe functioning. Additionally, it should also be noted that the TMT was not significantly correlated with performance on the RFFT, with the exception of the significant correlation between performance on the TMT Part A and the total number of unique designs produced on the RFFT. Taken together, the results suggest that the FTMT may be a measure of right frontal executive functioning.

Additional support for the sensitivity of the FTMT to right frontal lobe functioning is provided by the finding of a significant negative correlation between performance on Part D of the FTMT and high beta magnitude. We have previously used QEEG to provide neurophysiological validation of the RFFT [65] and the Rey Auditory Verbal Learning Test [70] and the present findings provide further support for the use of QEEG in validating neuropsychological tests. The lack of significant correlations between the TMT and either low or high beta magnitude may be related to a restricted range of scores on the TMT. As a whole, performance on the FTMT was more variable than performance on the TMT and this relatively restricted range for the TMT may have impacted the obtained correlations. Given the present findings, together with those of the Foster et al. [65, 70] investigations, further support is also provided for the use of EEG in establishing neurophysiological validation for neuropsychological tests.

The results from the factor analysis provide support for the contention that the FMT may be a measure of right frontal lobe activity and also provide initial discriminant validity for the FTMT. Specifically, Parts C and D of the FTMT were found to load on the same factor as the number of designs generated on the RFFT, although the time required to complete Part A of the TMT is also included. Additionally, the number of errors committed on Parts C and D of the FTMT comprises a single factor, separate from either the TMT or the RFFT. Although these results support the FTMT as a measure of nonverbal executive functioning, it would be helpful to conduct an additional factor analysis including additional measures of right frontal functioning, and perhaps other measures of right hemisphere functioning as marker variables.

We sought to develop a measure sensitive to right frontal lobe functioning due to the paucity of such tests and the potentially important uses that right frontal lobe tests may have clinically. Tests of right frontal lobe functioning may, for instance, be useful in identifying and distinguishing left versus right frontotemporal dementia (FTD). Research has indicated that FTD is associated with cerebral atrophy at the right dorsolateral frontal and left premotor cortices [71]. Fukui and Kertesz [72] found right frontal lobe volume reduction in FTD relative to Alzheimer’s disease and progressive nonfluent aphasia. Some have suggested that FTD should not be considered as a unitary disorder and that neuropsychological testing may aid in differentially diagnosing left versus right FTD [73].

Whereas right FTD has been associated with more errors and perseverative responses on the Wisconsin Card Sorting Test (WCST), left FTD has been associated with significantly worse performance on the Boston Naming Test (BNT) and the Stroop Color-Word test [73]. Razani et al. [74] also distinguished between left and right FTD in finding that left FTD performed worse on the BNT and the right FTD patients performed worse on the WCST. However, as noted earlier, the WCST has been associated with left frontal activity [9], right frontal activation [8], and bilateral frontal activation [7]. Further, patients with left frontal tumors perform worse than those with right frontal tumors [11].

Patients with FTD that predominantly involves the right frontotemporal region have behavioral and emotional abnormalities and those with predominantly left frontotemporal region damage have a loss of lexical semantic knowledge. Patients, in whom neural degeneration begins on the left side, often present to the clinicians at an early stage of the disease due to the presence of language abnormalities, but maintain their emotion processing abilities, being preserved the right anterior temporal lobe. However, as this disease advances, the disease may progress to the right frontotemporal regions. Tests sensitive to right frontal lobe functioning may be useful tools to identify in advance the course of the disease, providing immediate and specific treatments and informing the caregivers on the possible prospective frame of the disease.

A potentially more important use of tests sensitive to right frontal lobe functioning, though, may be in predicting dementia patients that will develop significant and disruptive behavioral deficits. Research has found that approximately 92 % of right-sided FTD patients exhibit socially undesirable behaviors as their initial symptom, as compared to only 11 % of left-sided FTD patients [75]. Behavioral deficits in FTD are associated with gray matter loss at the dorsomedial frontal region, particularly on the right [76].

Alzheimer’s disease (AD) is also often associated with significant behavioral disturbances. Even AD patients with mild dementia are noted to exhibit behavioral deficits such as delusions, hallucinations, agitation, dysphoria, anxiety, apathy, and irritability [77]. Indeed, Shimabukuro et al. [77] found that regardless of dementia severity, over half of all AD patients exhibited apathy, delusions, irritability, dysphoria, and anxiety. Delusions in AD patients are associated with relative right frontal hypoperfusion as indicated by SPECT imaging [78, 79]. Further, positron emission tomography (PET) has indicated that AD patients exhibiting delusions exhibit hypometabolism at the right superior dorsolateral frontal and right inferior frontal pole [80].

Although research clearly implicates right frontal lobe dysfunction in the expression of behavioral deficits, data from neuropsychological testing are not as clear. Negative symptoms in patients with AD and FTD have been related to measures of nonverbal and verbal executive functioning as well as verbal memory [81]. Positive symptoms, in contrast, were related to constructional skills and attention. However, Staff et al. [78] failed to dissociate patients with delusions from those without delusions based on neuropsychological test performance, despite significant differences existing in right frontal and limbic functioning as revealed by functional imaging. The inclusion of other measures of right frontal lobe functioning may result in improved neuropsychological differentiation of dementia patients with and without significant behavioral disturbances. Further, it may be possible to predict early in the disease process those patients that will ultimately develop behavioral disturbances with improved measures of right frontal functioning. Predicting those that may develop behavioral problems will permit earlier treatment and will provide the family with more time to prepare for the potential emergence of such difficulties. Certainly, future research needs to be conducted that incorporates measures of right and left frontal lobe functioning in regression analyses to determine the plausibility of such prediction.

Tests sensitive to right frontal lobe functioning may also be useful in identifying more subtle right frontal lobe dysfunction and the cognitive and behavioral changes that follow. The right frontal lobe mediates language melody or prosody and forms a cohesive discourse, interprets abstract communication in spoken and written languages, and interprets the inferred relationships involved in communications. Subtle difficulties in interpreting abstract meaning in communication, comprehending metaphors, and even understanding jokes that are often seen in right frontal lobe stroke patients may not be detected by the family and may also be under diagnosed by clinicians [82]. Further, patients with right frontal lobe lesions are generally more euphoric and unconcerned, often minimizing their symptoms [82] or denying the illness, which may delay referral to a clinician and diagnosis.

Attention deficit hyperactivity disorder (ADHD) is a neurological disease characterized by motor inhibition deficit, problems with cognitive flexibility, social disruption, and emotional disinhibition [83, 84]. Functional MRI studies reveal reduced right prefrontal activation during “frontal tasks,” such as go/no go [85], Stroop [86], and attention task performance [87]. The right frontal lobe deficit hypothesis is further supported by structural studies [88, 89]. Tests of right frontal lobe functioning may be useful in further characterizing the nature of this deficit and in specifying the likely hemispheric locus of dysfunction.

To summarize, we feel that right frontal lobe functioning has been relatively neglected in neuropsychological assessment and that many uses for such tests exist. Our intent was to develop a test purportedly sensitive to right frontal functioning that would be easy and quick to administer in a clinical setting. However, we are certainly not meaning to assert that our FTMT would be applicable in all the aforementioned conditions. Additional research should be conducted to determine the precise clinical utility of the FTMT.

Further validation of the FTMT should also be undertaken. Establishing convergent validation may involve correlating tests measuring the same domain, such as executive functioning. This was initially accomplished in the present investigation through the significant correlations between the TMT and the FTMT. Additionally, convergent validation may also involve correlating tests that purportedly measure the same region of the brain. This was also initially accomplished in the present investigation through the significant correlations between the FTMT and the RFFT. However, additional convergent validation certainly needs to be obtained, as well as validation using patient populations and neurophysiological validation.

We are currently collecting data that hopefully will provide neurophysiological validation of the FTMT. Certainly, though, it is hoped that the present investigation will not only stimulate further research seeking to validate the FTMT and provide more comprehensive normative data, but also stimulate research investigating whether the FTMT or other measures of right frontal lobe functioning may be used to predict patients that will develop behavioral disturbances.
